# Mental health and life satisfaction in East and West Germany: Effects of generation and migration of citizens

**DOI:** 10.3389/fpubh.2022.1000651

**Published:** 2022-11-29

**Authors:** Manfred E. Beutel, Lisa Braunheim, Ayline Heller, Gabriele Schmutzer, Oliver Decker, Elmar Brähler

**Affiliations:** ^1^Department of Psychosomatic Medicine and Psychotherapy, University Medical Center of the Johannes Gutenberg University Mainz, Mainz, Germany; ^2^Integrated Research and Treatment Center Adiposity Diseases, Behavioral Medicine Research Unit, Leipzig, Germany; ^3^Department of Social Psychology, Else-Frenkel-Brunswik-Institute University Leipzig, Sigmund-Freud-University Berlin, Berlin, Germany; ^4^Department of Psychiatry and Psychotherapy, University of Leipzig Medical Center, Leipzig, Germany

**Keywords:** mental health, internal migration, distress, life satisfaction, resilience, self-esteem, East/West Germany

## Abstract

**Objective:**

The reunification of Germany after the separation between 1949 and 1990 has offered a unique chance of studying the impact of socialization, political transformation, and migration on mental health. The purpose of this article was to compare mental distress, resources, and life satisfaction (1) between residents of East and West Germany and migrants who have fled from East to West Germany before reunification and (2) between three generations.

**Methods:**

We assessed anxiety, depression, resilience, self-esteem, and life satisfaction, comparing groups based on their residency and migration, as well as three different birth cohorts. Using a representative survey of 2006, analyses of variance show the differences between these groups. Based on a representative survey (*N* = 4,530), the effects of gender (53.6% women), residency/migration (74.4% grown up in the West, 20.4% in the East, 5.3% migrants from the East to the West) from three generations (32% born until 1945, 39% until 1967, and 29% to 1989), and positive and negative mental health indicators were analyzed.

**Results:**

Women reported higher distress and lower resilience. Residents of the Western states reported the lowest burden of distress (depressive and anxiety symptoms) and the highest overall life satisfaction, exceeding residents from the Eastern states and migrants from the Eastern to the Western states. Migrants from the Eastern to the Western states, however, reported the lowest resilience and self-esteem. They reported lower satisfaction with income, living conditions (compared to the Western residents), and the lowest levels of satisfaction with family (compared to East and West).

**Conclusion:**

Overall, our data point to inequalities between the Eastern and Western states regarding mental health 16 years after reunification favoring the residents of the Western states by lower distress and life satisfaction. Our data attest to the stresses and adjustments associated with migration from the Eastern to the Western states before reunification. A lower level of mental health and life satisfaction in the oldest generation may be related to the sequelae of World War II and also to aging.

## Introduction

The reunification of Germany after the separation between 1949 and 1990 has offered a unique chance of studying the impact of socialization, political transformation, and migration on mental health. After the Second World War (WWII), the two parts of Germany were rebuilt in different and antagonistic political systems. While the Federal Republic of Germany (FRG), today's old federal states plus West Berlin, was rebuilt after the model of the Western allies, the German Democratic Republic (GDR) followed the socialist model. Conditions of socialization and living drifted apart between the two German states, and specific risk and protective factors for mental health evolved corresponding to the specific experiences in the two political systems. According to socialist ideology in particular women and lower classes were integrated into the labor force minimizing experiences of unemployment and reducing inequality. Development of qualification, continuing education, and child and healthcare could have acted as protective factors in the GDR, whereas social inequality and traditional gender roles in the FRG may have been risk factors for mental health for women and lower classes. On the other hand, experiences of political repression and incarceration have been shown to induce physical complaints, anxiety, and depression in the GDR.

The “fall of the Berlin wall” and the subsequent formal accession of the GDR to the FRG brought immense economic, cultural, and political transformation. Despite large monetary transfer to rebuild the new states, the former East German population suffered from the loss of social and vocational security: several million inhabitants lost their jobs (1), and many moved to the West (2) and the remaining population often perceived themselves as second class citizens (3). Results regarding mental health indicators, however, were scarce and mixed. A large nationally representative survey reported higher prevalences of mental disorders in the Western states in 1998/9 (4) concerning substance abuse, depression, somatoform disorders, social anxiety, and eating disorders. Brähler et al. (5) found participants residing in the Eastern states reported more physical symptom load. In a large representative survey with 5,036 participants in 2006, male and female participants from the Eastern states reported higher levels of anxiety and depression than those from the Western states (6–8). Negative effects of transformation (e.g., extremely high rates of previously unknown unemployment in the Eastern state in the course of economical reorganization) and persistent economic disadvantages may have compromised mental health in the Eastern states. In the meantime, living conditions, and general and mental health have also converged. Depression was even lower in the Eastern states in 2019 (9), and recently, the younger generation fared better under pandemic conditions in the Eastern than in the Western states (10).

In previous surveys, we have shown that mental health has shifted between generations who grew up under different living conditions. Particularly those born before 1945 have often been exposed to bombing, expulsion from their homes, loss of their primary caretakers (in particularly their fathers), hunger, and other atrocities of World War II. As we have found, adverse war experiences have been associated with increased anxiety and depression many decades after the end of WWII (11).

Migration is considered a process of more or less permanent and distant relocation, usually between different countries, often fraught with multiple losses, uncertainties, and perils in the process. Additional adjustment required to the different cultural, legal, economic, and political system has been termed acculturation. Learning new behaviors, customs, and languages and adjusting to new social demands often becomes a major life stress with different adaptive (integration and assimilation) or maladaptive outcomes (12). A substantial proportion of the German population was subjected to life-threatening experiences of flight or expulsion before 1946. Fifty-seven years after the end of WWI, those afflicted reported worse quality of life, health, and wellbeing and suffered from increased panic attacks (11). Based on a total of about three million people, a large proportion of the East German population has subsequently fled to West Germany from 1948 to 1989 (13). Motives ranged from escape from political oppression, restricted freedom of movement, education or choice or persecution, and the wish to reunite with family members living in West Germany to economical motives due to higher living standards in the West. The vast majority fled illegally, which was heavily persecuted by incarceration, losses of social contacts, property, social status, and careers of those caught and their next to kin. Following the construction of the border wall to prevent flight in 1961, more and more refugees were arrested, injured, or killed in the process. Following reunification, large proportions of the population of the Eastern states resettled in the Western states, and only recently, the proportions of those moving from the Eastern to the Western states and vice versa have become balanced. For the latter groups, a healthy migrant effect can also be postulated, that is, physically and mentally healthy citizens seek migration.

In 2006, we compared a sizable representative sample of three groups as follows: those residing in the Eastern states of Germany, in the Western states of Germany, and those migrating from Eastern to Western Germany. We distinguished the following three cohorts: (a) born until 1945, the end of World War II, (b) and the decades between 1946 and 1967, as well as (c) and between 1968 and 1989, the fall of the wall between GDR and FRG.

We assessed anxiety and depression as the most common indicators of mental distress in the general population by the standardized self-report scales of the PHQ-2 for depression (14) and the Generalized Anxiety Scale Gad-7 (8). Additionally, we assessed resilience (15) and self-esteem (16) as mental health resources, along with life satisfaction (17).

The purpose of this article was to compare mental distress, resources, and life satisfaction (1) between residents of East and West Germany and migrants who have fled from East to West Germany before reunification and (2) between the three generations (born before 1945, 1946–1967, and 1968–1989).

## Materials and methods

### Study design and participants

The data used in this article are from the representative Health Survey by the University of Leipzig Medical Center, and a face-to-face household survey conducted in 2006 in Germany. They originate from a private database. Within this survey, the study participants were interviewed using a structured self-report questionnaire. The commercial survey institute USUMA (Independent Service for Survey, Methods, and Analysis) collected the data over two waves.

Using a multistage sampling to randomly select a representative for Germany in regards to sociodemographic variables, 258 sample point regions throughout all German regions were drawn from the most recent political election register. In the second step, a random route procedure led to the selection of the households, followed by a Kish selection of household members over the age of 14.

Eight thousand one hundred six of the 8,398 addresses were valid. Trained personnel interviewed participants at their homes. All participants gave their informed consent. After sociodemographic questions, self-report questionnaires regarding health status and political attitudes were answered. Here, help was given only upon the direct request of the respondents. The representativeness of the sample was confirmed by the German market research institutes association Arbeitskreis Deutsche Marktforschungsinstitute (ADM).

Surveys were carried out in accordance with the Declaration of Helsinki; further, they met the ethical guidelines of the International Code of Marketing and Social Research Practice of the ICC (International Chamber of Commerce) and the ESOMAR (European Society of Opinion and Marketing Research). Every researcher and the member concerned with the collection of the data are committed to the guidelines of good scientific practice. They affirmed participants in their self-determination and confirmed confidentiality. Of note, 1,199 persons (14.8%) of the 8,106 valid addresses were not at home at the three attempted visits of the interviewers and 1,806 persons refused to participate (22.3%). Because of severe illness, 65 participants (0.8%) could not complete the survey. Finally, a total of 5,036 persons agreed to participate and completed the study, providing verbal informed consent. Thus, the response rate was 72.9% (5,036/6,907), whereas participation the rate among all eligible subjects was 62.1% (5,036/8,106).

For the following analyses, *N* = 299 participants were excluded as they had grown up abroad (migrants). Due to the small number (*N* = 39) of internal migrants, persons who had moved from the West to the East were excluded. The same was true for participants born after 1989 (*N* = 101), as these were only aged 14–17 at the time of the survey and hardly comparable to the adult population.

The demographic characteristics of our study population (*N* = 4,530) are summarized in Table 1, according to those who had grown up in the West (74.4%), in the East (20.4%), and those who migrated from the East to the West (5.3%).

**Table 1:** Sample description by residency.

	**East**	**East/West**	**West**	**Total**
	***N* = 924** ** (20.4%)**	***N* = 240** ** (5.3%)**	***N* = 3,376** ** (74.5%)**	***N* = 4,530**
**Sex**				
Male	431 (46.6)	119 (49.6)	1,527 (45.2)	2,077 (45.7)
Female	493 (53.4)	121 (50.4)	1,849 (54.8)	2,463 (54.3)
**Generation**				
Before 1945	331 (35.8)	64 (26.7)	1,046 (31.0)	1,441 (31.7)
1946–1967	339 (36.7)	81 (33.8)	1,361 (40.3)	1,781 (39.2)
1968–1989	254 (27.5)	95 (39.6)	969 (39.6)	1,318 (29.0)
**Partnership**				
Yes	570 (61.7)	130 (54.2)	2,042 (60.5)	2,742 (60.4)
No	354 (38.3)	110 (45.8)	1,334 (39.5)	1,798 (39.6)
**A-Level education**				
Yes	153 (16.6)	38 (15.8)	450 (13.3)	641 (14.1)
No	771 (16.6)	202 (84.2)	2,926 (86.7)	3,899 (85.9)
**Equivalized income (**€**)**				
M	1,147 (467)	1,344 (645)	1,521 (719)	1,435 (719)

We present absolute numbers; numbers in brackets are percentages, respectively standard deviation (income).

The mean (SD) age of the participants was 48.4 (18.0) years. More than 50% were women in the three subsamples (total 54.3%). Comparable proportions were born until 1945 (total 32%), until 1967 (39%), respectively until 1989 (29%). The youngest generation was slightly underrepresented in the group from the Eastern states. Approximately 60% had a partnership in the population of the Eastern and Western states (slightly lower in migrants with 54%). The level of education was highest in East and lowest in West Germans, the income was reversed with the highest income in those living in the Western and lowest in those in the Eastern states.

### Assessments

The survey questionnaire included questions about age, gender, education, marital status, employment status, net household income, nationality, place of residence, and church membership.

Resilience is measured by the 11-item short form (RS-11) validated by Schumacher et al. (15). It is conceptualized as a protective personality factor that is associated with a healthy development and psychosocial stress resistance, using a 7-point Likert scale “from ‘1' = strongly disagree to ‘7' = strongly agree” (Cronbach's alpha = 0.91). The RS-11, conceptualized as a unidimensional scale, has shown to be a reliable and valid instrument that allows an economic assessment of resilience in a community sample (18).

The valid PHQ-2 includes the first two items of the PHQ-9 which are originally part of the DSM-IV (Diagnostic and Statistical Manual of Mental Disorders) Diagnostic Criterion A symptoms for major depressive disorder (“feeling down, depressed, or hopeless”; “little interest or pleasure in doing things”). The scales vary between “not at all”, “several days”, “more than half the days”, and “nearly every day”, scored as 0, 1, 2, and 3, respectively, using a four-point Likert scale (Cronbach's alpha = 0.78) (19).

The GAD-7 identifies probable cases of generalized anxiety disorder and further assesses symptom severity. It was validated for both care patients and the general population. The seven items describe the most prominent diagnostic features of the DSM-IV diagnostic criteria A, B, and C for generalized anxiety disorder. The answers rank between “not at all”, “several days”, “more than half the days”, and “nearly every day”, scored as 0, 1, 2, and 3, respectively, using a four-point Likert scale (Cronbach's alpha = 0.89) (8).

The German Adaptation of Rosenberg's Self-Esteem scale (RSES) was administered. The RSES consists of five positively (e.g., “I am satisfied with myself.”) and five negatively worded items (e.g., “At times, I think I am not good at all.”). The answers vary between four response categories, which are “from ‘0' = strongly agree to ‘3' = strongly disagree”, using a 4-point Likert scale. Psychometric properties of the scale are well documented, including Cronbach's α = 0.88 (16).

The life satisfaction scale (FLZM) reflects aspects of a general sense of wellbeing. The questions on life satisfaction (FLZM) are a multi-dimensional self-report measure of general life satisfaction and satisfaction with health with established international normative data (17). The general domains cover friends, leisure time activities/hobbies, general health, income, profession, housing/living conditions, family life, and partnership/sexuality. Respondents rate their present satisfaction with these dimensions on a scale from “1 = dissatisfied” to “5 = very satisfied,” using a 5-point Likert scale (Cronbach's α = 0.88).

### Data analysis

Data analyses were performed by parametric and non-parametric tests as appropriate. We compared mean somatic symptoms across surveys *via* 2 × 3 × 3 Analysis of variance with the three factors gender (male and female), residency/migration (East/East, East/West, and West/West), and generation (born till 1945, 1946–1967, and 1968–1989) with subsequent Scheffe tests to compare the mean differences. As no adjustments for multiple testing were made, we consider our findings exploratory. We computed analyses using SPSS 24 (Statistical Package for the Social Sciences). For all analyses, we specified an error probability of α < 0.05. We used listwise deletion for missing data.

## Results

**Figure 1:**
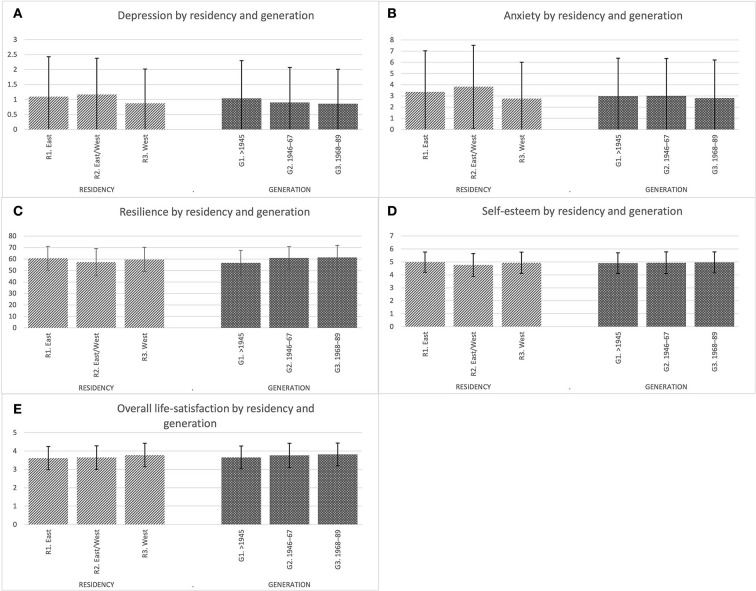
**(A)** Depression by residency and generation. **(B)** Anxiety by residency and generation. **(C)** Resilience by residency and generation. **(D)** Self-esteem by residency and generation. **(E)** Overall life-satisfaction by residency and generation. Presented are means and SDs. ANOVA (Scheffe-tests with *p* < 0.05 in parentheses for all groups). M, male; F, female; R, residency; G, generation. **(A)** *N* = 4,517. (1) Sex *F*_(l/4,499)_ = 12.53; *p* < 0.001; (2) Residency *F*_(2/4,499)_ = 17.98; *p* < 0.001 (R1, R2 > R3); (3) Generation *F*_(2/4,499)_ = 9.00; *p* < 0.001 (G2, G3 > G1); (4) Sex*Generation *F*_(2/4,499)_ = 9.15; *p* < 0.001. **(B)** *N* = 4,537. (1) Sex *F*_(l/4,519)_ = 27.22; *p* < 0.001; (2) Residency *F*_(2/4,519)_ = 21.75; *p* < 0.001 (R1, R2 > R3); (3) Generation *F*_(2/4,519)_ = 1.90; *p* = 0.15; (4) Sex*Generation *F*_(2/4,519)_ = 3.68; *p* < 0.05. **(C)** *N* = 4,527. (1) Sex *F*_(1/4,509)_ = 5.07; *p* < 0.05; (2) Residency *F*_(2/4,509)_ = 14.52; *p* < 0.001 (R1 < R2, R3; R3 > R2); (3) Generation *F*_(2/45,09)_ = 95.04; *p* < 0.01 (G2, G3 > GI); (4) Sex*Residency *F*_(2/4,509)_ = 3.04; *p* < 0.05; (5) Sex*Generation *F*_(2/4,509)_ = 9.84; *p* < 0.001. **(D)** *N* = 4,527. (1) Sex *F*_(1/4,509)_ = 0.54; *p* = 0.462; (2) Residency *F*_(2/4,509)_ = 7.82; *p* < 0.001 (R1, R3 > R2); (3) Generation *F*_(2/4,509)_ = 2.66; *p* = 0.071); (4) Residency*Generation *F*_(2/4,509)_ = 2.45; *p* < 0.05. **(E)** *N* = 4,532. (1) Sex *F*_(1/4,514)_ = 0.13; *p* < 0.717; (2) Residency *F*_(2/4,514)_ = 31.16; *p* < 0.01; (3) Generation *F*_(2/4,514)_ = 23.45; *p* < 0.001; (4) Sex*Generation *F*_(2/4,514)_ = 11.35; *p* < 0.001.

As Figures 1A,B shows that depressive and anxiety symptoms were higher in women compared to men and in Eastern residents and in East to West migrants compared to Western residents. Additionally, more depressive symptoms were reported by the oldest (war) vs. the younger generations. As indicated by significant interactions in sex by generation, however, only the oldest women had increased scores compared to subsequent generations: (born before 1945: depression women M = 1.20; men 0.86, *p* < 0.001; anxiety women M = 3.32; men 2.58; *p* < 0.05).

Regarding resilience, as can be seen in Figure 1C, men reported slightly higher scores than women. The highest resilience was reported by Eastern compared to Western residents. East/West migrants had the lowest resilience. Across generations, resilience was lowest in the oldest compared to the two younger groups. As indicated by interactions, women in the oldest group reported the lowest resilience (*p* < 0.001), and women in the East/West migrants group also reported the lowest resilience (*p* < 0.05) compared to residents from the Western, respectively Eastern states.

Figure 1D shows that men and women did not differ regarding self-esteem. Lower self-esteem was reported by East–West migrants compared to Eastern and Western residents. As indicated by interaction (*p* < 0.05), the lowest self-esteem was reported by East and West migrants from the generation born from 1946 to 1967.

Figure 1E portrays that overall life satisfaction was not affected by gender. However, life satisfaction was higher in the Western population compared to those in the East and East/West migrants. Regarding generations, the oldest one reported the lowest overall life satisfaction compared to the middle and the youngest generations. Again, life satisfaction was lowest among women in the oldest generation (*p* < 0.001).

Table 2 shows the single dimensions of life satisfaction, according to gender, residency, and cohort. Women reported higher satisfaction with friendships, partnership, sexuality, and family life, whereas men were more satisfied with leisure time and health. Satisfaction with specific dimensions was highest in those residing in the Western states regarding income, friendships, leisure time activities, and housing. Interestingly, migrants from East to West Germany reported lower satisfaction with income, and housing and the lowest levels of satisfaction with family (compared to East and West). There was no difference regarding the partnership. Satisfaction with income was higher in women among Eastern residents but otherwise higher in men. Satisfaction with friendships was lowest in the oldest group and highest in the youngest group. Women in the youngest group were least satisfied. Regarding family life, the middle age group was most satisfied. While in the oldest group men were more satisfied, women were more satisfied in the younger groups. Men in the oldest groups had higher scores and women in the younger groups. Satisfaction with partnership and sexuality was lowest in the oldest group. As the interaction indicates, the lowest scores were found for women in the oldest and the highest for women in the youngest group. The lowest satisfaction was found in male migrants and the highest in male West Germans. Satisfaction with leisure time was highest in the youngest group, and the highest satisfaction was found in men in the West (interaction). Satisfaction with housing, work, and health was lowest in the youngest group. Men in the west were most satisfied with their profession (M = 3.61), whereas men and women in the East least (M = 3.07). Health satisfaction was highest in men from the West.

**Table 2:** Participants' dimensions of life satisfaction by sex, generation, and residency.

	**M**	**Income**	**Friendships**	**Family life**	**Partnership**	**Leisure time**	**Housing**	**Profession**	**Health**
Sex	Female	3.40 (1.02)	3.96 (0.78)	4.00 (0.98)	3.59 (1.17)	3.71 (0.85)	4.00 (0.83)	3.45 (1.06)	3.78 (0.95)
	Male	3.41 (1.03)	3.90 (0.79)	3.86 (0.92)	3.68 (1.09)	3.79 (0.82)	3.98 (0.83)	3.48 (1.13)	3.82 (0.93)
	Significance *p*	0.750	0.011^*^	0.000^***^	0.003^**^	0.001^**^	0.389	0.399	0.042^*^
Residency	East	3.09 (1.08)	3.85 (0.83)	3.93 (0.96)	3.62 (1.20)	3.63 (0.85)	3.97 (0.76)	3.07 (1.24)	3.67 (0.99)
	East/West	3.21 (1.01)	3.91 (0.80)	3.73 (1.08)	3.54 (1.17)	3.69 (0.86)	3.79 (0.91)	3.39 (1.13)	3.80 (0.83)
	West	3.51 (0.98)	3.96 (0.77)	3.95 (0.94)	3.64 (1.11)	3.78 (0.83)	4.01 (0.84)	3.58 (1.02)	3.83 (0.93)
	Significance *p*	0.00^**^	0.001^**^	0.007^**^	0.204	0.000^***^	0.001^**^	0.049^*^	0.000^***^
Generation	Before 1945	3.50 (0.88)	3.83 (0.79)	3.94 (0.89)	3.31 (1.14)	3.70 (0.84)	4.04 (0.78)	3.40 (1.05)	3.42 (0.94)
	1946 – 67	3.40 (1.05)	3.90 (0.78)	4.00 (0.95)	3.77 (1.09)	3.71 (0.83)	4.04 (0.82)	3.48 (1.11)	3.80 (0.91)
	1968 – 89	3.31 (1.11)	4.09 (0.75)	3.85 (1.01)	3.78 (1.12)	3.85 (0.85)	3.86 (0.89)	3.51 (1.11)	4.20 (0.81)
	Significance *p*	0.000^***^	0.00^**^	0.000^***^	0.00^**^	0.000^***^	0.000^***^	0.00^**^	0.00^**^

* *p* < 0.05,

** *p* < 0.01,

*** *p* < 0.001. Standard deviations are given in brackets.

## Discussion and implications

Our data show that mental health was strongly related to sex, residency, respectively migration, and generation. As in previous analyses, women reported higher distress (8) and lower resilience than men (18). They reported higher satisfaction with friendships, partnership, sexuality, and family life, whereas men were more satisfied with leisure time and health. Residents of the Western states reported the lowest burden of distress (depressive and anxiety symptoms) and the highest overall life satisfaction, exceeding both residents from the Eastern states and migrants from the Eastern to the Western states. Life satisfaction in residents of the Western states was highest regarding friendships, leisure time activities, income, and living conditions. Residents from the Eastern states reported the highest resilience. Migrants from the Eastern to the Western states, however, reported the lowest resilience and self-esteem. They reported lower satisfaction with income, living conditions (compared to the Western residents), and the lowest levels of satisfaction with family (compared to East and West).

Regarding generation, the highest depression scores were reported by the oldest generation who also reported the lowest resilience and overall life satisfaction compared to the two younger generations. Satisfaction with specific dimensions was highest in those residing in the Western states regarding income, friendships, leisure time activities, and housing. Interestingly, migrants reported lower satisfaction with income, and housing and the lowest levels of satisfaction with family (compared to East and West). There was no difference regarding the partnership.

Regarding age, satisfaction with friendships, partnership, sexuality, housing, profession, and health were lowest in the oldest group. Satisfaction with leisure time and health was highest in the youngest group.

Overall, our data point to inequalities between the Eastern and Western states regarding mental health 16 years after reunification favoring the residents of the Western states by lower distress and life satisfaction. Higher life satisfaction in the Western states extended to the majority of, but not all dimensions of quality of life: income, friendships, leisure time activities, health, work, and housing. No differences were found regarding family, partnership, and sexuality. These discrepancies may partly reflect the effects of the ongoing transformation of the Eastern states. A lower level of income (Table 1), high rates of unemployment, respectively premature pension decrease satisfaction with income and with work. Recently, we could show that wealth is predictive of life satisfaction (20). The still-increasing wealth gap clearly acts in favor of the wealthier inhabitants of the Western states.

Interactions of residency with gender reflect different living conditions of women and men in former FRG and GDR: Higher satisfaction with income in female Eastern (compared to Western) residents may reflect the fact that a gender pay gap favoring men was and still is a reality in Western, but not former Eastern Germany. Similarly, the high satisfaction with work in Western men compared to women and the comparatively low satisfaction in Eastern men and women may reflect both, the challenge of the transformation with subsequent job losses and shifts, and the male Western role of professional achievement.

Our data shed light on the understudied issue of migration from GDR to FRG. This is a special case of migration, as GDR and FRG were ruled by antagonistic political systems, but shared the language and cultural heritage. Following reunification, migration could be considered simply relocation within one country. Heightened distress in the group of East–West migrants can be considered a consequence of stresses in the premigration process (e.g., political incarceration), the migration process (e.g., mortal danger), and the postmigration (acculturation) process. Their comparatively low satisfaction with family life may also be a consequence of family disruption due to the flight. Lower satisfaction with income and living conditions compared to West German residents may indicate that these migrants see themselves as disadvantaged in West Germany. Consistent with the Interactive Acculturation Model (21), these findings attest to the stresses and adjustments associated with migration from the Eastern to the Western states before reunification. These may have eroded resilience.

A commonality in participants growing up in the GDR and FRG, respectively the migrants, was that mental health was also affected by generation. Those born until 1945, who had experienced the atrocities of World War II, reported worse mental health than the younger generations. Specific decrements in life satisfaction such as leisure time, partnership, or health may be due to the loss of functional capacities and social contacts due to bereavement. Loss of a partner may particularly affect women due to their longer life expectancy as reflected in the lowest rating of partnership and sexuality. As in previous studies, we found that the female gender was associated with more self-reported distress, in participants from Eastern and Western Germany and across generations. Gender effects were most prominent in the oldest generations, who have probably shared the most traditional gender roles in Germany.

“Both effects” considered the effects of socialization in the separated regions as well as the effect of transformation. Recently, we could show that there were subsequent shifts in reported mental distress between the former Eastern and Western states (22). We recently investigated internal migrants again. Their heightened mental distress compared to residents in the Eastern states underlines persistent acculturation stress even when voluntarily and freely moving from the Eastern to the Western states (23). Future studies should not only be limited to larger scale comparisons of regions as in our study but also attend to the significance of smaller scale regional differences (24).

## Limitations

The strengths of the study were the large sample size and the broad array of mental health indicators, including distress, resources, and life satisfaction. We excluded migrants born in other countries, as these were presumed to be a very heterogeneous group in the various migration waves since WWII (12).

We do not know when participants migrated from GDR to FRG, and we do not know their motives (e.g., if they were traumatized by previous incarceration and political persecution). While we had the unique chance to study generation, socialization, migration, and cohort effects on mental health, we are aware that over the past 16 years, mental health has shifted between Eastern and Western states, and there has been a historical change of aging processes and conceptions in midlife which was likely to also affect symptom reporting (25).

## Resource identification initiative

All analyses were carried out using SPSS (RRID:SCR_002865).

## Data availability statement

The raw data supporting the findings of this article will be made available by the corresponding author upon reasonable request.

## Ethics statement

Ethical review and approval was not required for the study on human participants in accordance with the local legislation and institutional requirements. Written informed consent to participate in this study was provided by the participants' legal guardian/next of kin.

## Author contributions

MB wrote the main manuscript text. AH edited the manuscript. LB prepared figures and tables. GS prepared the analyses. OD and EB supervised. All authors reviewed the manuscript. All authors contributed to the article and approved the submitted version.

## Funding

This research was supported by the German Federal Ministry of Education and Research (Grant Number 01UJ1911AY).

## Conflict of interest

The authors declare that the research was conducted in the absence of any commercial or financial relationships that could be construed as a potential conflict of interest.

## Publisher's note

All claims expressed in this article are solely those of the authors and do not necessarily represent those of their affiliated organizations, or those of the publisher, the editors and the reviewers. Any product that may be evaluated in this article, or claim that may be made by its manufacturer, is not guaranteed or endorsed by the publisher.
